# The ability to increase the base of support and recover stability is limited in its generalisation for different balance perturbation tasks

**DOI:** 10.1186/s11556-021-00274-w

**Published:** 2021-10-05

**Authors:** Jil Bosquée, Julian Werth, Gaspar Epro, Thorben Hülsdünker, Wolfgang Potthast, Kenneth Meijer, Rolf Ellegast, Kiros Karamanidis

**Affiliations:** 1 Sport and Exercise Science Research Centre, School of Applied Sciences, London South Bank University, 103 Borough Road, SE1 0AA London, UK; 2 Department of Exercise and Sport Science, LUNEX International University of Health, Exercise and Sports, Differdange, Luxembourg; 3 Institute of Biomechanics and Orthopedics, German Sport University Cologne, Cologne, Germany; 4 Department of Nutrition and Movement Sciences, NUTRIM School of Nutrition and Translational Research in Metabolism, Maastricht University Medical Centre+, PO Box 616, 6200 MD Maastricht, The Netherlands; 5 Institute for Occupational Safety and Health of the German Social Accident Insurance (IFA), Sankt Augustin, Germany

**Keywords:** Tripping, Falls, Gait perturbation, Reactive stepping, Lean-and-release test, Dynamic stability

## Abstract

**Background:**

The assessment of stability recovery performance following perturbations contributes to the determination of fall resisting skills. This study investigated the association between stability recovery performances in two perturbation tasks (lean-and-release versus tripping).

**Methods:**

Healthy adults (12 young: 24 ± 3 years; 21 middle-aged: 53 ± 5 years; 11 old: 72 ± 5 years) were suddenly released from a forward-inclined position attempting to recover stability with a single step. In a second task, all participants experienced a mechanically induced trip during treadmill walking. To assess dynamic stability performance, the antero-posterior margin of stability (MoS), the base of support (BoS), and the rate of increase in BoS were determined at each foot touchdown (TD) for both tasks.

**Results:**

Only weak to moderate correlations in dynamic stability performance parameters were found between the two tasks (0.568 > *r* > 0.305, 0.001 < *p* < 0.04). A separation of participants according to the number of steps required to regain stability in the lean-and-release task revealed that multiple- (more than one step) compared to single-steppers showed a significantly lower MoS at TD (*p* = 0.003; *g* = 1.151), lower BoS at TD (*p* = 0.019; *g* = 0.888) and lower rate of increase in BoS until TD (*p* = 0.002; *g* = 1.212) after release. Despite these profound subgroup differences in the lean-and-release task, no differences between multiple- and single-steppers were observed in the stability recovery performance during tripping.

**Conclusion:**

The results provide evidence that the ability to effectively control dynamic stability following a sudden balance disturbance in adults across a wide age range is limited in its generalisation for different perturbation tasks.

## Introduction

Daily-life locomotion is a challenging task. While walking on slippery or uneven paths, crossing over obstacles lying on the ground or managing to pass along narrow walkways, one faces countless situations that can disturb movement, requiring the neuromotor system to adjust its motor strategies to cope with external perturbations (e.g. a trip), control stability or avoid falls. Although falls are observed among adults of all ages, their incidence increases with aging contributing to the most prominent cause for injuries, hospitalization or even death among the elderly population [1–3]. Therefore, assessing and understanding stability recovery mechanisms in adults of various ages is highly relevant to reduce or even avoid forward falls and related injuries at old age [4–6].

To maintain stability during walking, the central nervous system needs to ensure a continuous interaction between perceptual information and motor responses [7]. Human locomotion requires the combination of multiple sensory information originating from somatosensory, vestibular, and visual systems, together with the coordination of numerous skeletal muscles. When experiencing an unexpected trip during locomotion, a change in the relation between the centre of mass (CoM) and the base of support (BoS) is observed, with the CoM moving closer to the edge of the BoS. This change leads to a significant decrease in the margin of stability (MoS) compared to unperturbed walking, causing an instable body configuration [8, 9]. Hence, in order to increase the MoS and efficiently counteract a forward fall, a relatively long and rapid anterior step is required [4]. Given that older as well as middle-aged compared to younger adults require on average more steps to regain a stable MoS following a sudden stability loss [10, 11], large focus has been placed on developing testing paradigms to evaluate stability recovery mechanisms following sudden stability loss.

Various studies have investigated human stability recovery performance and the ability to increase effectively the BoS in the anterior direction following externally induced stability perturbations using an unexpected release from a forward inclined position, i.e. the lean-and-release task [6, 8, 12–15]. Previous research has demonstrated that future fall risk in older populations can be predicted by the recovery stepping behaviour observed in such lean-and-release tasks [12]. Süptitz and colleagues [16] reported that following a sudden gait-trip perturbation, older in comparison to young adults show a decreased capacity to rapidly and effectively increase their BoS, indicating to a higher fall risk. This could explain why older adults often require multiple steps to regain their stability during trip-like perturbations.

The ability to increase effectively the anterior BoS is an essential skill to regain stability control in a lean-and-release task [17] as well as during tripping [6, 18]. Besides, it has been reported that a significant increase in BoS of the recovery step following an anterior stability loss in both tasks can be observed when compared to unperturbed walking [8]. Although critical task parameters (e.g. muscle activity patterns, muscle-tendon-unit lengths and body dynamics) may differ possibly due to different body configurations, and the static or dynamic nature, both tasks involve perturbations being large enough to cause instable body configurations which require similar stability control mechanisms (i.e. increase in anterior BoS due to rapid stepping) crucial for safe locomotion and fall prevention in everyday life. Thus, one might suggest a link between the reactive stepping performances in these tasks. Regarding this, a recent study showed no inter-task transfer of fall-resisting skill adaptations from short-term treadmill gait-perturbation exercise to a lean-and-release task [8], suggesting only a limited generalisation of improved fall-resisting skills. Nevertheless, the mentioned study focused on the transfer of adaptations acquired during a gait-perturbation exercise on the treadmill to a lean-and-release task, rather than on an association of stability recovery performance between the two tasks. Up to date, literature is still lacking information regarding the association of the capability to regain stability effectively and rapidly between the lean-and-release task and tripping-task in adults of various age. This could be of great interest for clinical settings regarding the evaluation of dynamic stability performance in aging adults.

Therefore, the present study aimed to examine the relationship between the stability recovery performance during lean-and-release task and a tripping-task on a treadmill among adults across a wide age range (*n* = 44; 24 to 72 years). In addition, it was investigated whether there are differences in treadmill tripping performances between single- and multiple-steppers observed in the lean-and-release task. It was hypothesized that stability recovery during a lean-and-release task is not a valid measure to appropriately predict tripping recovery performance.

## Methods

### Participants and experimental design

A total of 44 healthy adults of various ages (24–72 years) participated in this study. Participants were not eligible to perform the experiments if they were suffering from any movement limiting neurological or musculoskeletal impairments or diseases of the lower limbs. After an initial briefing, all participants provided their informed consent. In the first stability recovery task, all participants were unexpectedly released from a static forward-inclined position (lean-and-release task). Following this, they were exposed to an unexpected trip-like perturbation while walking on a treadmill at a given speed. To ensure safety, participants were secured by a full-trunk safety harness attached to an overhead track allowing antero-posterior and medio-lateral movements but preventing any contact of the body with the ground (except for the feet). The present study was approved by the ethics committee of the German Sport University Cologne (ethical approval no. 141/2017) and was conform to all requirements for human experimentation in accordance with the Declaration of Helsinki.

### Lean-and-release task

Participants’ stability recovery performance was evaluated using a lean-and-release task, that has been described in previous studies [8, 17]. Briefly, the participants were standing on a force plate (1080 Hz, 60 × 90 cm: Kistler, Winterthur, Switzerland) with their feet in parallel and flat on the ground. They were gradually inclined in the forward direction and held by a custom-built pneumatic break-and-release system via a horizontally running inextensible Teflon cable connected to a belt around the pelvis [10]. The targeted inclination matched an angle corresponding to a value of 23 ± 2% body weight and was controlled with the means of a load cell implemented in series with the supporting cable. The exact forward lean was chosen according to previous results of the reduced ability of older adults to regain balance within a single recovery step from cable loads of more than 23% body weight [17]. Once any anticipatory movement was attenuated (i.e. antero-posterior and medio-lateral weight shift corrections, checked real-time via cable load and ground reaction forces) the supporting cable was released without any further notice after an arbitrary period between 10 and 30 s. Prior to the measurement, participants were previously instructed to try regaining a stable stance with a single recovery step after being released using the limb of their choice [19]. To guarantee novelty of the task, no prior practice trials were performed.

**Fig. 1:**
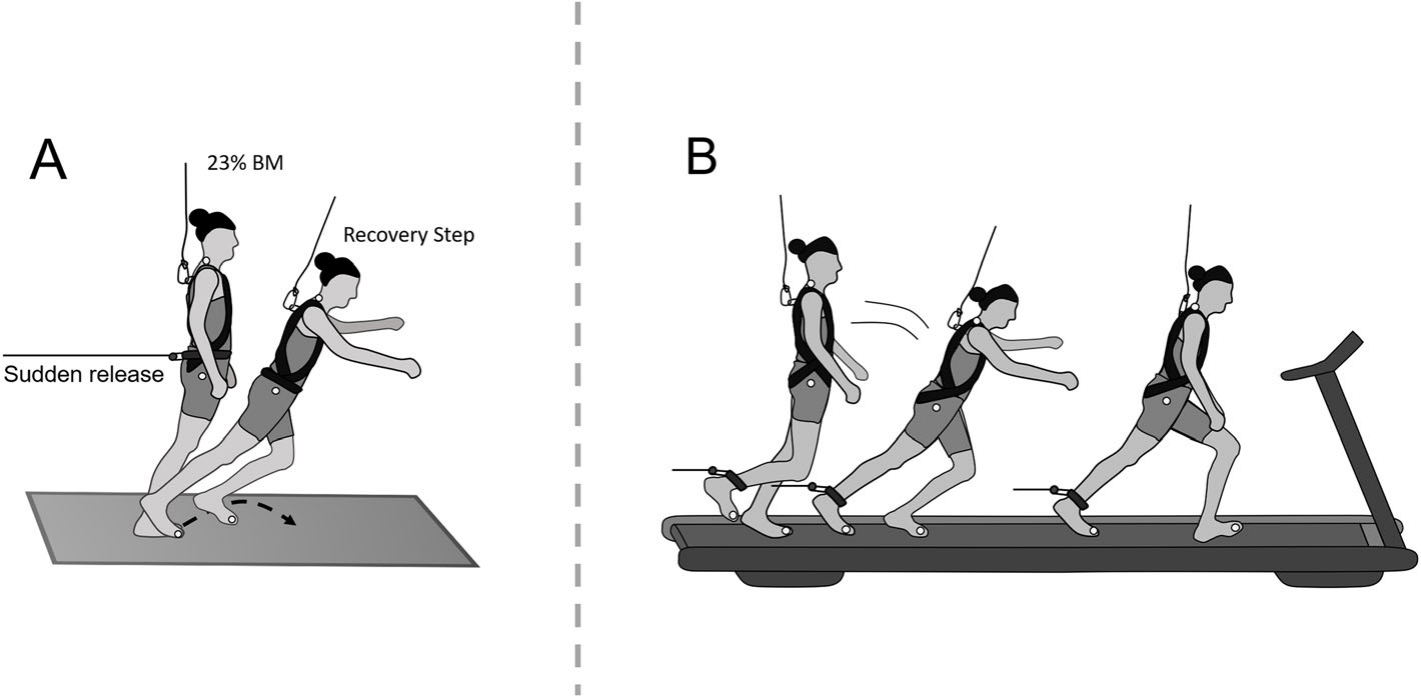
Schematic illustration of the experimental set-ups. **A** Lean-and-release-task. Participants were released once from a forward-inclined position**.** Lean angles were normalized to the participants’ body weight (23% of body weight) ensuring to standardize the level of stability loss. **B** Tripping-task during treadmill walking. Participants were exposed to a trip while walking on a treadmill. The trip was induced using a manually monitored custom-built pneumatic brake-and-release system. In the event of a fall, an overhead safety harness prevented the participant’s body (except the feet) from touching the treadmill belt. White circles represent the five retroreflective markers attached to anatomical landmarks used to evaluate the spatiotemporal stepping characteristics during both tasks

According to previous findings [10], stability recovery performance was categorised into two stepping behaviours, i.e. single stepping versus multiple stepping. Participants were defined as single-steppers if they needed only one step to recover stability or if a follow-up step of the contralateral limb did not exceed the anterior displacement of the recovery limb’s foot. Consequently, multiple stepping was identified if participants required any additional step of the recovery limb or needed a safety harness support, i.e. more than 20% of body weight observed via a second load cell integrated into the harness suspension cable [20] (Fig. 1).

### Single exposure to a trip-like perturbation during treadmill walking

The tripping-task used in the current study has been conducted previously [8, 18]. The protocol started with the participants walking unperturbed on a treadmill (pulsar 4.0; h/p/cosmos, Nussdorf-Traunstein, Germany) at a standardised speed of 1.4 m/s for 4 min followed by a baseline measurement (25 stride cycles of walking). Subsequently, they were exposed to an unexpected trip-like perturbation induced using a custom-built pneumatic perturbation system and encouraged to continue walking afterwards. Throughout one entire swing phase, the perturbation (restraining pull) was applied using a strap attached to the right ankle connected via a Teflon cable to the perturbation device. Although participants received prior information about the task, they were not able to anticipate the onset and removal of the perturbation. All participants were invited to familiarise only with unperturbed treadmill walking 4–7 days prior to the measurement day. To guarantee novelty of the task, no exposures to treadmill perturbations were performed prior to the actual measurement.

### Data collection and processing

To determine the CoM trajectories and dynamic stability control during the two tasks, a reduced kinematic model was used [16]. Five retroreflective markers were attached to anatomical landmarks (seventh cervical vertebra, both greater trochanters and forefeet of the left and right legs, respectively) and tracked via a 10-camera optical motion capture system (120 Hz; Nexus 2.6.1; Vicon Motion Systems, Oxford, UK). Three-dimensional coordinates of the markers were smoothed using a fourth-order digital Butterworth filter with a cut-off frequency of 20 Hz [18]. Foot touchdown (TD) of the recovery step in the lean-and-release task was determined as the moment at which the vertical ground reaction force measured by a second force plate (1080 Hz, 60x90cm; Kistler) exceeded a threshold value of 20 N. For the tripping task, TD was defined as the impact peak of an analogue signal acquired using 2-D accelerometers (±50 g, 1080 Hz; model ADXL250; Analog Devices, Norwood, MA) positioned on the tibia of each leg [16]. The antero-posterior margin of stability (MoS) was calculated as the difference between the anterior boundary of the base of support (BoS) and the extrapolated centre of mass (XCoM), which includes both the position and the velocity of the CoM. The MoS and BoS were assessed at each TD during unperturbed, perturbed, and the first six recovery steps following the perturbation [18], as well as at TD of the first recovery step during the lean-and-release task [17]. The BoS was calculated as the distance between the toe markers of the trailing and stance limb at TD for both tasks. Furthermore, the rate of increase in BoS during the lean-and-release task was calculated as the ratio between the BoS at TD and the swing time until TD of the first recovery step.

### Statistics

Normal distribution of all variables was confirmed by Lillifors-corrected Kolmogorov-Smirnoff tests (*p* > 0.05). To examine the relationship between the lean-and-release task and the tripping task performance across participants, Pearson product-moment correlation coefficients were computed for the MoS, the BoS, and the rate of increase in BoS. Since younger adults are not representative of high fall risk, subgroup comparisons (single-steppers versus multiple-steppers) regarding dynamic stability during the lean-and-release task as well as during the tripping-task were performed including only middle-aged and older adults. Independent samples *t*-tests were used to examine differences between single-steppers and multiple-steppers in the MoS, the BoS, and the rate of increase in BoS for the lean-and-release task. Subgroup comparisons for the tripping-task were performed using separate two-way mixed-measures ANOVAs with factors subgroups (single- versus multiple-steppers) and events (perturbed and the following six recovery steps) for the MoS and the BoS. In case of significant main effects or interactions, Duncan’s post-hoc corrections were applied. The level of significance was set at α = 0.05 and effect sizes were calculated using Hedges’g and partial eta square $\left({\eta}_p^2\right)$. Effect sizes were considered small (${\eta}_p^2$ =0.01; *r* = 0.1; g = 0.2), medium (${\eta}_p^2$ =0.06; *r* = 0.3; g = 0.5), or large (${\eta}_p^2$ =0.14; *r* = 0.5; g = 0.8). To identify age-related differences in the MoS, the BoS, and the rate of increase in BoS amongst the three age-groups (young, middle-aged, old) during the lean-and-release task, separate one-way ANOVAs were used. Separate two-way mixed-measures ANOVAs were used to detect age-related differences in the MoS, and the BoS during the tripping-task, with age-group (young, middle-aged, old) and events (perturbed and the following six recovery steps) as factors. Differences in age, body height and mass as well as physical activity between the three age groups were analysed using separate one-way ANOVAs. In cases of significant main effects or interactions, Duncan’s post-hoc tests were applied. All statistical and non-statistical analyses were performed using Statistica software (Release 10.0; Statsoft Inc., Tulsa, OK, USA) and MATLAB (2020b, MathWorks®, Natick, MA, USA).

## Results

### Association of stability recovery performance between lean-and-release task and tripping

**Fig. 2:**
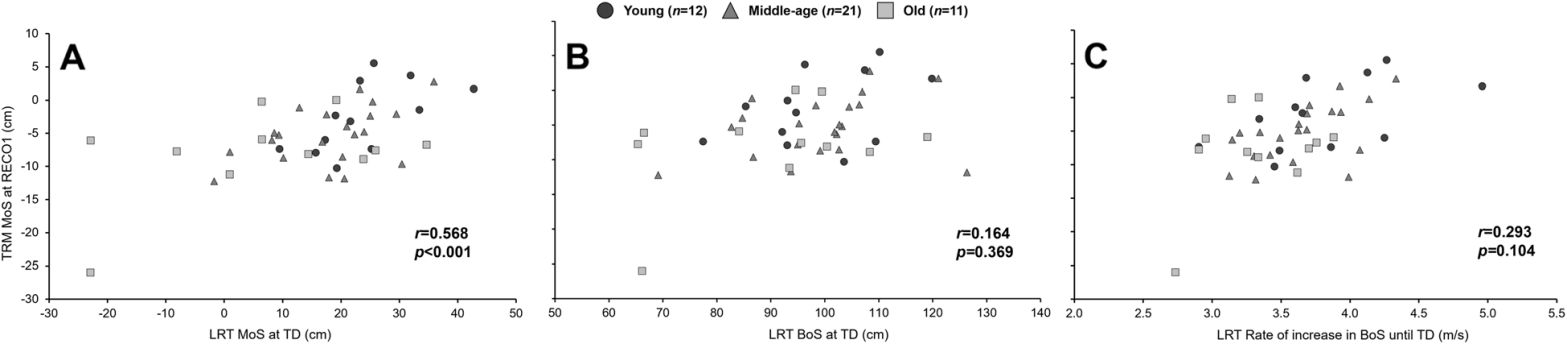
**A** Relationship between the margin of stability (MoS) of the first recovery step of the tripping task (TRM) and the MoS at foot touchdown (TD) during the lean-and-release-task (LRT). **B** Relationship between the MoS of the first recovery step of the TRM task and the base of support (BoS) at foot TD during the LRT. **C** Relationship between the MoS of the first recovery step of the TRM task and the rate of increase in BoS until foot TD during the LRT

**Fig. 3:**
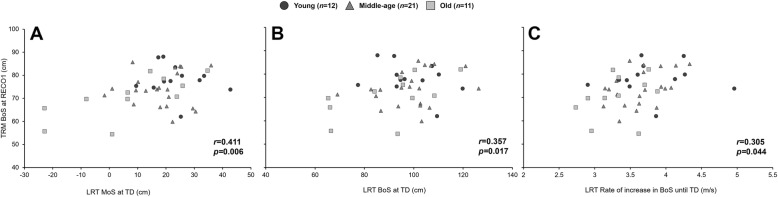
**A** Relationship between the base of support (BoS) of the first recovery step of the tripping task (TRM) and the margin of stability (MoS) at foot touchdown (TD) during the lean-and-release-task (LRT). **B** Relationship between the BoS of the first recovery step of the TRM task and the BoS at foot TD during the LRT. **C** Relationship between the BoS of the first recovery step of the TRM task and the rate of increase in BoS until foot TD during the LRT

There were statistically significant correlations between stability recovery performances (MoS and BoS at TD and rate of increase in BoS until TD) of the lean-and-release task and the tripping-task (MoS and BoS at TD of the first recovery step). Although significant, weak to moderate correlations were found between the MoS at TD during tripping and the MoS at TD of the lean-and-release task (*r*_44_ = 0.568, *p* < 0.001; Fig. 2) as well as between the BoS at TD during tripping and the BoS at TD during the lean-and-release task (*r*_44_ = 0.305, *p* = 0.044; Fig. 3). Similarly, there was a significant correlation between the BoS at TD of the lean-and-release task and its respective rate of increase in BoS until TD (*r*_44_ = 0.600, *p* < 0.001). Furthermore, a significant correlation was detected between the BoS at TD during tripping and both the MoS at TD (*r*_44_ = 0.411, *p* = 0.006; Fig. 3) as well as the rate of increase in BoS at TD (*r*_44_ = 0.357, *p* = 0.017; Fig. 3) of the lean-and-release task. No statistically significant correlations was found between the MoS at TD during tripping and both the BoS at TD, or the rate of increase in BoS until TD during the lean-and-release task (Fig. 2).

### Single- and multiple-stepper subgroup comparison

**Fig. 4:**
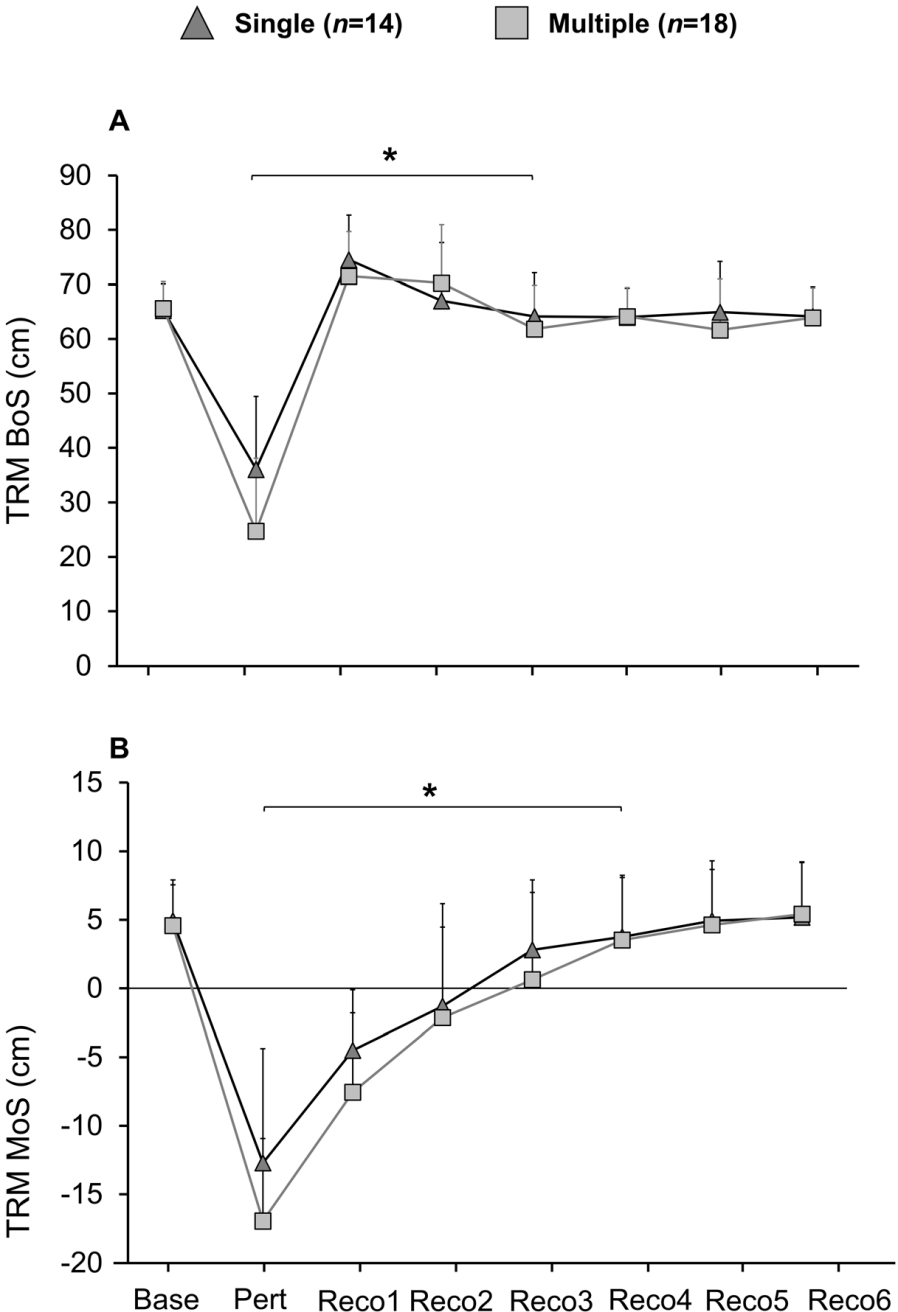
**A** Base of support (BoS) and **B** margin of stability (MoS) during the tripping task (TRM) for single- (*n* = 14) and multiple-steppers (*n* = 18). Data is shown for baseline walking (Baseline), at touchdown (TD) of the perturbation (Pert) as well as for the 6 recovery steps following the perturbation (RECO1-RECO6) for the two subgroups. Values are presented as means with SD error bars. *: significant different BoS (first three recovery steps) and MoS (first four recovery steps) when comparing two consecutive steps (*p* < 0.001)

Eighteen out of 44 participants were determined as multiple-steppers following sudden stability loss in the lean-and-release task (none of the young, 40% of the middle-aged and 90% of the old adults). Since younger adults are not representative of high fall risks, only middle-aged and older adults were included in the subgroup comparisons [single-steppers (*n* = 14) versus multiple-steppers (*n* = 18)] for dynamic stability control. Multiple- compared to single-steppers showed significantly lower MoS at TD [t (30) = 3.228, *p* = 0.003, *g* = 1.151], lower BoS at TD [t (30) = 2.49, *p* = 0.019, *g* = 0.888], as well as lower rates of increase in BoS until TD [t (30) = 3.352, *p* = 0.002, *g* = 1.212] during the lean-and-release task, with no significant differences in the MoS at release. There were no significant differences between multiple- and single-steppers in the MoS as well as the BoS at TD accounting for the steps from perturbation to the sixth recovery step during tripping (Fig. 4). There was a statistically significant event-effect in the MoS and BoS of consecutive steps [F (6,180) = 150.408, *p* < 0.001, ${\eta}_p^2$ =0.834; F (6,180) = 105.152, *p* < 0.001, ${\eta}_p^2$ =0.778] independent of the subgroups. Post-hoc analysis revealed a higher MoS in the first four recovery steps (*p* < 0.001) and a significantly higher BoS in the first three recovery steps (*p* < 0.001), when comparing one step to the following one.

### Age-related effect on stability recovery performance

**Fig. 5:**
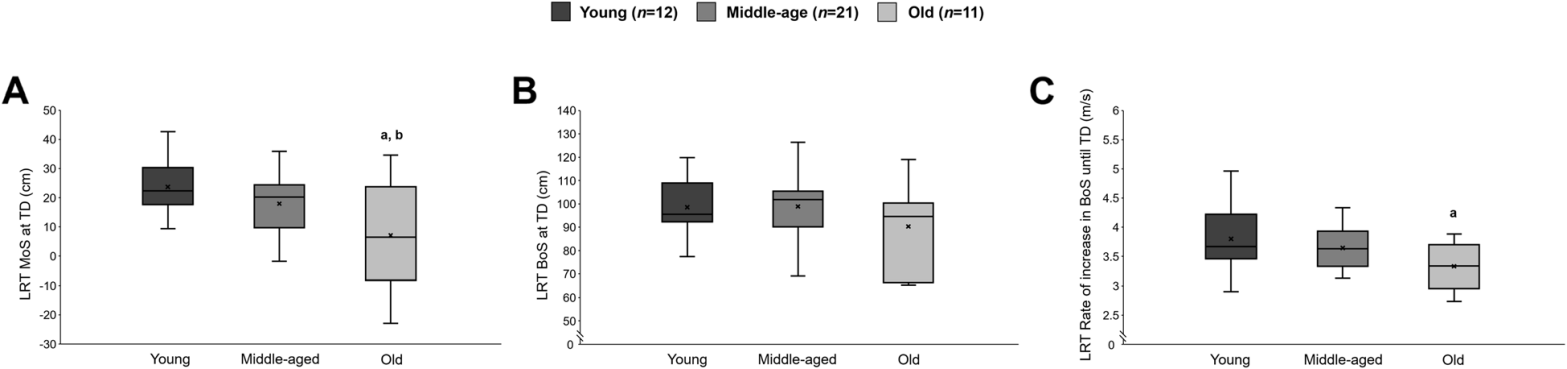
**A** Base of support (BoS) and **B** margin of stability (MoS) at foot touchdown (TD) and the **C** rate of increase in BoS until foot TD during the lean-and-release task (LRT). Results are presented as boxplots with the mean (line), median (x) and interquartile range between 25th and 75th percentile along with minimum and maximum values for all three age-groups [young (*n* = 12), middle-aged (*n* = 21) and older adults (*n* = 11)]. a: old statistically different to young (0.002 < *p* < 0.007); b: old statistically different to middle-aged (*p* = 0.03)

There was a statistically significant effect in age [F (2,41) = 312.42, *p* < 0.001, ${\eta}_p^2$ =0.934] between the three analysed groups: young: 24 ± 3 years; middle-aged: 53 ± 5 years; older: 72 ± 5 years. Body height (176 ± 8 cm vs. 173 ± 11 cm vs. 170 ± 9 cm), body mass (70.8 ± 11.6 kg vs. 74.8 ± 12.7 kg vs. 73.3 ± 12.8 kg) and physical activity (6.2 ± 2.4 h/week vs. 6.6 ± 4.6 h/week vs. 6.5 ± 2.5 h/week) did not significantly differ between the three age groups. Regarding the MoS at TD of the recovery step in the lean-and-release task, there was a significant age effect [F (2,41) = 5.279, *p* = 0.009, ${\eta}_p^2$ = 0.205; Fig. 5], with older adults showing a lower MoS compared to young (*p* = 0.002) and middle-aged (*p* = 0.028) adults (Fig. 5). The rate of increase in BoS showed a statistically significant age effect [F (2,41) = 3.896, *p* = 0.028, ${\eta}_p^2$ =0.159], with lower rates of increase in BoS for older compared to young adults (*p* = 0.007; Fig. 5). No differences between groups in the BoS at TD were found.

**Fig. 6:**
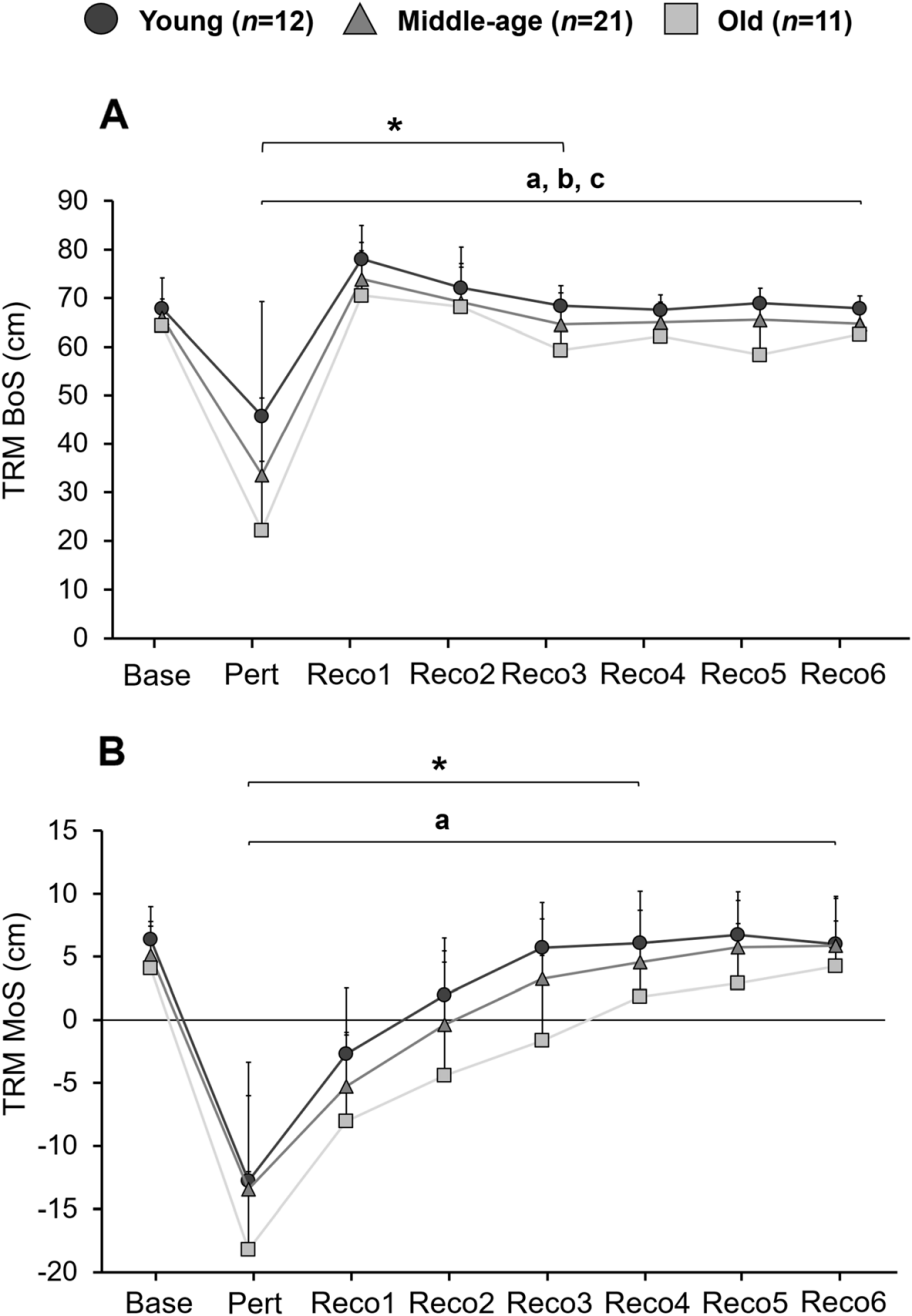
**A** Base of support (BoS) and **B** margin of stability (MoS) during tripping task (TRM). Data is shown for baseline walking (Baseline), for touchdown (TD) at perturbation (Pert) as well as for the 6 recovery steps following the perturbation (RECO1-RECO6), in young (*n* = 12), middle-aged (*n* = 21) and older adults (*n* = 11). Values are presented as means with SD error bars. a: old significantly different to young (0.001 < *p* < 0.008); b: old significantly different to middle-aged (*p* = 0.011); c: middle-aged significantly different to young (*p* = 0.013); *: significant different BoS (first three recovery steps) and MoS (first four recovery steps) when comparing two consecutive steps (*p* < 0.001)

Following the applied trip-like perturbation while walking, the MoS at TD of the perturbed step was on average − 12.8 ± 9.4, − 13.5 ± 7.5 and − 18.2 ± 6.1 cm for young, middle-aged, and older adults, respectively. The analysis of the MoS at TD of the perturbed and following six recovery steps revealed a significant age effect [F (2,41) = 3.74, *p* = 0.030, ${\eta}_p^2$ =0.154]. Post-hoc tests revealed that older compared to young adults had a significantly (*p* = 0.008) lower MoS at TD (Fig. 6). Although not reaching a statistical significance, there was a tendency (*p* = 0.053) for a lower MoS at TD during tripping in older compared to middle-aged adults. Additionally, there was a significant event effect [F (6,246) = 196.35, *p* < 0.001, ${\eta}_p^2$ =0.827] in the MoS at TD. Post-hoc analysis revealed a significantly higher MoS at TD in the first four recovery steps, when comparing two consecutive steps (*p* < 0.001). Regarding the BoS at TD, there were significant age [F (2,41) =11.75, *p* < 0.001, ${\eta}_p^2$ =0.364] and event [F (6,246) = 101.93, *p* < 0.001, ${\eta}_p^2$ =0.713] effects following the trip. Post-hoc analysis for age revealed a lower BoS at TD in older compared to the young (*p* < 0.001) and middle-aged adults (*p* = 0.011). Furthermore, middle-aged compared to young adults showed a lower (*p* = 0.013) BoS at TD following the trip (Fig. 6). Post-hoc analysis of the event-effect showed a significantly higher BoS at TD for the first three recovery steps, when comparing two consecutive steps (*p* < 0.001).

## Discussion

The present study aimed to examine the association between the stability recovery performances in a lean-and-release task and a tripping-task during treadmill walking among adults of various ages. In addition, it was investigated if separating the participants into subgroups according to their stability recovery behaviour in the lean-and-release task (single- vs. multiple-steppers) reveals differences between the subgroups’ recovery behaviour in the tripping-task. Whilst there were significant correlations between the lean-and-release and the tripping task, those were mainly weak to moderate with only up to one third of explained variance and heterogeneous in terms of statistical significance. Moreover, despite clear differences in the lean-and-release performance, single- and multiple-steppers demonstrated similar stability control when recovering from tripping. The combined pattern of results hence indicates limited generalisation of stability recovery performance between both tasks.

Previous research has shown that stability recovery performance in a lean-and-release task is a good predictor of future fall risk among older adults [12]. The current study revealed similar to earlier studies [12, 14, 15] a gradual age-related deterioration in the ability to recover stability with a single rapid step following a sudden stability loss as well as diminished recovery performance in tripping. It is widely accepted that the ability to increase the BoS rapidly and effectively in the anterior direction is an essential component of dynamic stability control [21] and represents one main mechanisms to recover stability following a sudden forward fall [17] or trip [6, 18]. Since this mechanism is evoked in a similar manner in a lean-and-release task and during tripping, an association of the stability recovery performances between the two tasks could be expected. The current study however revealed no consistent pattern in the results, with only some of the correlations showing significant but weak correlations, indicating that the changes in MoS and BoS of the tripping-task are not related to the stepping behaviour of the lean-and-release task (Fig. 2). Moreover, comparisons between single- and multi-steppers indicated differences only in stability recovery performance during the lean-and-release task but not in tripping recovery during walking on the treadmill. Despite an enhanced ability to rapidly increase the BoS and control stability in single- compared to the multiple-steppers during the lean-and-release task, there were no group-related differences in the recovery performance following a trip-like perturbation (BoS, MoS at TD from perturbed and the following six recovery steps). Thus, correlations and subgroup analyses did not indicate a functionally relevant association in stability recovery performances between both tasks.

Even though an important attribute of the neuromotor system is the capacity to transfer skills from one task to another, up to date literature is still lacking knowledge regarding the topic of inter-task transfer. It is suggested that the lean-and-release task and the tripping-task share similar stability recovery mechanisms [8], i.e. to increase the BoS rapidly and effectively in the anterior direction. To support this task similarity, an additional analysis for the BoS at TD during baseline walking, first recovery step during tripping as well as during the lean-and-release task was performed. Results showed significantly higher (*p* < 0.001) values in the first recovery step for the tripping-task and lean-and-release task compared to baseline walking (BoS at TD during baseline walking: 66 ± 5 cm; gait perturbation: 74 ± 8 cm; lean-and-release task: 97 ± 14 cm; *p* < 0.001). This confirms previous observations stating that an effective anteriorly increase in the BoS is required to recover stability following a sudden large perturbation as in the two tasks investigated, hence strengthening the assumption of a shared stability recovery mechanism [8]. However, the present study was unable to prove functionally relevant associations between the stability recovery performance of both tasks, suggesting that stepping recovery in a lean-and-release task seems not to be a valid measure to predict the recovery performance after tripping. These results are supported by earlier findings reporting no inter-task transfer of fall-resisting skills from an unexpected trip-perturbation to a sudden release from a forward-inclined position [8]. In contrast, previous studies found positive transfers of adaptations between different tasks using similar perturbation methods, i.e. slipping evoked by platform translation to untrained walking over a slippery surface [22]. These opposing findings suggest that generalisation of stability recovery skills from one task to another might be possible but seem to be limited if factors beyond common recovery mechanism differentiate perturbation responses in motor tasks sharing the same main stability recovery mechanism.

Previous research has shown that different biomechanical demands or perturbations elicit distinct ‘task-specific’ motor components, even between highly similar tasks, e.g. mechanically induced perturbations during standing on a stable or unstable platform [23, 24]. Despite that in the current study the MoS at TD of the perturbed step during treadmill tripping (on average for all analysed subjects: − 0.15 ± 0.09 m) matched the MoS at the time point of release during the lean-and-release task (− 0.14 ± 0.08 m), differences in task difficulty cannot be ruled out entirely as a contributing factor to the low correlations. Although sharing a similar stability control mechanism, the absolute values of the magnitude of increase in the BoS were approximately 1.3 times higher for the lean-and-release task compared to the tripping task, which may have at least partly been induced by the lean-and-release task being more challenging due to its task-specific requirement to regain stability using a single step. Nevertheless, the current findings revealed significant age-related differences in recovering from tripping, whereas multiple- versus single-steppers in the lean-and-release task demonstrated no differences during tripping. Thus, whilst both tasks clearly demonstrated challenges on dynamic stability control, limited transfer and generalisation cannot be explained only based on the weak or moderate inter-task correlations but further on the sub-group comparisons (single vs. multiple steppers). Although not in the scope of the current study, a possible explanation for the lack of generalisation for recovery performances to different perturbation tasks may lay beyond the similarities in spatiotemporal stepping characteristics. Thus, it cannot be excluded that neuromotor control required to recover stability might differ between the two deployed tasks in the current study, i.e. they share only a limited number of muscle synergies possibly affecting the small associations between recovery performances. Regarding this, although the lean-and-release as well as tripping task are frequently considered for the investigation of stability recovery performance, they should not be used interchangeably in clinical settings.

It is important to note that the current study addressed only the antero-posterior components of dynamic stability control since both tasks consist of anteriorly induced perturbations. One might argue that the medio-lateral stability could have played a role potentially affecting the current results. However, when analysing spatiotemporal components (medio-lateral directed increase in BoS and velocity of the CoM at TD), both parameters were in absolute terms multiple factors lower than the antero-posterior components for each task respectively (increase in BoS on average during LRT and TRM for the medio-lateral versus antero-posterior direction: 0.01 ± 0.06 m versus 0.97 ± 0.14 m, and 0.05 ± 0.15 m versus 0.74 ± 0.08 m; velocity of the CoM at TD on average: 0.19 ± 0.45 m/s versus 1.28 ± 0.22 m/s, and 0.12 ± 0.06 m/s versus 1.36 ± 0.17 m/s, respectively). Thus, we are confident that the effects of the medio-lateral stability during the anteriorly directed perturbations used in the current study were less functionally relevant compared to the antero-posterior components.

## Conclusion

In conclusion, no functionally relevant associations were identified between the recovery performances following a sudden stability loss from a static forward-inclined position and a novel trip during treadmill walking. Moreover, similar to previously performed studies the current results showed deteriorations in the ability to recover from unexpected stability perturbations with aging. Thus, the current study provides evidence that the ability to increase the BoS and effectively recover from stability perturbations deteriorates with aging and is limited in its generalisation for different perturbation tasks in adults across a wide age range.

## Data Availability

The datasets used and/or analyzed during the current study are available from the corresponding author on reasonable request.

## Abbreviations

MoS: Margin of stability
BoS: Base of support
TD: Touchdown
CoM: Centre of mass
